# “It Bothered Me”: The Mental Burden of COVID-19 Media Reports on Community-Dwelling Elderly People

**DOI:** 10.3390/medicina59112011

**Published:** 2023-11-15

**Authors:** Natasa Maksimovic, Tatjana Gazibara, Jelena Dotlic, Marija Milic, Vida Jeremic Stojkovic, Smiljana Cvjetkovic, Gordana Markovic

**Affiliations:** 1 Institute of Epidemiology, Faculty of Medicine, University of Belgrade, 11000 Belgrade, Serbia; 2 Faculty of Medicine, University of Belgrade, 11000 Belgrade, Serbia; drenadot@gmail.com; 3 Clinic for Obstetrics and Gynecology, University Clinical Center of Serbia, 11000 Belgrade, Serbia; 4 Institute of Public Health of Serbia “Dr. Milan Jovanovic Batut”, 11000 Belgrade, Serbia; marijamilic85@gmail.com; 5 Department of Epidemiology, Faculty of Medicine, University of Pristina Temporarily Seated in Kosovska Mitrovica, 38220 Kosovska Mitrovica, Serbia; 6 Department of Humanities, Faculty of Medicine, University of Belgrade, 11000 Belgrade, Serbia; 7 Primary Health Care Center “Zemun”, 11000 Belgrade, Serbia

**Keywords:** COVID-19, reporting, information, processing, psychological burden

## Abstract

*Background and Objectives*: Elderly people may have difficulties understanding the quality and quantity of information about the COVID-19 epidemic, which can put an additional mental strain on their health and well-being. The purpose of this study was to explore the processing of COVID-19 information among older people. *Materials and Methods*: A qualitative study was carried out in summer 2021. The sampling was based on the snowball method. This approach allowed us to communicate with the next potential participants relatively freely and without reservations. Two female researchers (both MD, PhD) conducted the interviews. All interviews were held in Serbian. The data were analyzed using qualitative content analysis. *Results*: The interviews were conducted with 13 participants (average age 71 years). The analysis of qualitative content suggested that four topics could be identified: (1) sources of information, (2) information interest and need, (3) reporting of information and (4) suggestions for better reporting. The participants were troubled by the excess of information, repetitive information about death tolls, unqualified people in media discussing the pandemic and inconsistent reporting. These features caused the participants to feel the psychological burden in processing all the pieces of information. *Conclusions*: The elderly people in Serbia followed mainstream media to get information about COVID-19; however, they perceived a variety of problems with reporting, which made the understanding of the information difficult and psychologically burdensome. These findings should be taken into consideration when delivering health-related information to elderly people.

## 1. Introduction

Elderly people have been recognized as a high-risk group for poorer physical and mental health outcomes of COVID-19. The mental health burden among elderly people during the COVID-19 epidemic could have been additionally influenced by a so-called infodemic [1], a phenomenon specific to epidemics that arises from the overload of information that may not necessarily be accurate [2]. Erroneous information can be particularly troublesome for people who are not medical professionals. In fact, in the presence of unclear and confusing information, people are less likely to adhere to preventive behaviors and avoid exposure to the virus [3,4].

In the course of an outbreak such as COVID-19, elderly people are especially vulnerable because of social isolation, reduced mobility, limited finances and frailty. They may also be exposed to domestic abuse [5,6]. In these circumstances, elderly people may have difficulties understanding the quality and quantity of information about the epidemic, which can put an additional mental strain on their health and well-being [4]. As a result, they may exercise less caution and increase their risk of contracting the virus.

Thus far, few studies have focused on the experiences and processing of information related to the COVID-19 pandemic among elderly adults [7]. Because of this, there is a gap in the knowledge about the challenges that elderly people encountered regarding information appraisal and comprehension since the beginning of the COVID-19 pandemic. Such data are important to understand the information needs of elderly people and adjust or adapt information delivery in the future.

The purpose of this study was to explore the processing of information about the COVID-19 pandemic and understand potential challenges in handling such information among elderly people in Serbia.

## 2. Materials and Methods

A qualitative study was carried out in July and August 2021. The Ethics Committee of the Faculty of Medicine, University of Pristina temporarily seated in Kosovska Mitrovica approved the study (approval no. 2179/21). Before enrollment, participants provided a signed informed consent and agreed to the audio recording of the interviews.

The interviews were based on the thematic guide constructed by the study authors in order to focus on the major points of interest in this research (Table 1). The thematic guide was structured based on discussions with the experts and on the empirical evidence [8,9]. This thematic guide helped the interviewers to have sequence and flow during the interviews with study participants. It covered aspects that were relevant to the study’s aim and prevented the conversations from being diluted.

### 2.1. Selection of Study Participants

The sampling strategy to recruit the participants was based on the snowball method. This approach allowed us to communicate with the next potential participants relatively freely and without reservations. Additionally, because elderly people may be more vulnerable to exploitation due to their health status and isolation during the COVID-19 pandemic, a bond of trust between the researchers and the potential participants had to be established in advance [10]. We identified first 2 elderly persons (aged > 65) in 2 different cities in Serbia via face-to-face contact. All participants resided in an urban setting. The first 2 people represented the base from which we further contacted other participants following their recommendations.

Thirteen people were interviewed out of 15 who were invited to participate (response rate 86.7%). The two invited people were not willing to discuss the content. After having reached thematic saturation, the recruitment of the study participants was finalized. The point of reaching thematic saturation was discussed among the interviewers. This moment was identified when the researchers acknowledged that the addition of new participants provided repetitive narratives but no new insights.

Because all the participants were elderly people, we applied the Mini Mental State Examination to test their cognitive capacity (cutoff ≥ 24 points). None of the participants had any cognitive disturbances.

### 2.2. Procedure

The relationship with the participants was established before the interviews were scheduled via telephone call. This was done first through the person who recommended their participation and then when the researchers called to introduce themselves and the study. On this occasion, the study and the research aims were explained to the participants. A face-to-face interview was scheduled after receiving consent for participation and audio recording. The interviews took place at the participants’ homes. Only the researcher and the participant were in the room where the interviews took place.

Two female researchers (both MD, Ph.D.) with academic backgrounds and prior experience in theoretical and practical qualitative research conducted the interviews. The interviews were carried out only once. All interviews were held in Serbian. The interviews took approximately 20 min. After the audio recording, the researchers transcribed the interviews verbatim. After the process of transcription, the audio recordings were destroyed. The transcripts were not returned to the participants for comments.

### 2.3. Trustworthiness and Rigor

The researchers’ role in data collection and shaping of the data analysis was considered.

Elderly people are the population group who experienced an almost 2-month-long lockdown in Serbia [11] and faced various challenges due to isolation. Despite the fact that the majority of elderly people were in favor of lockdown as a means to control the pandemic, their social interactions were minimized as well as physical activity. As a result, they spent their time mostly listening to news about COVID-19. Because this study was conducted while the COVID-19 pandemic was ongoing and related information was actively broadcasted, participants’ accounts of media reporting were vivid and occasionally emotionally charged. The narratives of the study participants were consistent and frequently overlapped, suggesting that the experience of media reporting had a similar effect or was interpreted in a similar manner.

The interviewers, albeit younger than the study population, also lived through the lockdown and were informed through national and international media. For this reason, they were able to relate to the participants’ interpretation of the style of media reporting and further deepen the conversation to collect as many impressions and feedback as possible. This link allowed the interviewers to better understand the position of elderly people in Serbia in the midst of the COVID-19 pandemic.

### 2.4. Data Analysis

The collected data were analyzed using a qualitative content analysis because the purpose of this research was to gather as much descriptive data as possible [12]. Themes were not based on the thematic guide, even though there might be some overlap. Two researchers who conducted the interviews also performed the data analysis. The analysis included the reading of the transcripts line-by-line. Codes were assigned to each meaningful answer. Codes that had similar features and that logically related to one another were grouped together and adjusted as needed. The newly assigned codes were compared to the previous codes for comparison and meaning. No coding tree was made.

Coding was discussed with all members of the research team for clarity and consistency to make the classification of themes as accurate as possible. No software was used to for data management and data analysis. The content analysis was performed on transcripts in the Serbian language. The representative quotes were translated into English. The translation was verified by a native English speaker.

## 3. Results

### 3.1. Socio-Demographic Characteristics of Participants

Interviews were conducted with 13 elderly people. Of those, seven (53.8%) were women. The mean age of participants in this study was 71.3 ± 4.4 years (age range 67–80 years). The majority of the elderly people in our study had a higher education level (84.6%). 

### 3.2. Themes

The analysis of the qualitative content suggested that four themes could be identified: (1) sources of information, (2) information interest and need, (3) reporting of information and (4) suggestions for better reporting.

#### 3.2.1. Sources of Information

The elderly people in this study used various sources of information about COVID-19. All the people articulated that they followed the news on TV. Most people read newspapers as well. Some read information on the Internet, such as news articles, posts on social media, and scientific articles and official websites from institutions such as the World Health Organization. Some shared posts with their peers and family using smartphones. A few were in contact with their general practitioner.

“*Television, internet, colleagues…from all sides…I comment a little with my wife, this, that*…”

(Participant 1)

“*I got informed through media and Internet, I followed our press conferences every day*”

(Participant 3)

“*Well, television and radio. My wife was reading Internet posts. I did not. And, um…on television, since there are a lot of channels, you can catch everything live, from American to English…those informative things, you know*.”

(Participant 4)

The source of information was strongly related to trust in the accuracy of the information. All participants agreed that official sources of information, i.e., what was broadcasted by the media, was the main source of information about COVID-19. The interviewees were primarily interested in what government representatives and physicians (pulmonologists, epidemiologists and infectious diseases specialists) were discussing in the media, as they considered them the most reliable sources of information. They also compared the content from various sources and the information delivery by different people.

“*I had [trouble finding accurate information] at the beginning in the sense that I was not sure what information was trustworthy, the one from media, on the Internet, from newspapers, they did not match, but then I decided to trust only one source, or else I would have gone mad, I would get confused, I would become depressed… I decided to follow, I cannot say that I trusted it 100%, but I followed the official information*.”

(Participant 2)

#### 3.2.2. Interest and Need for Information

Some participants in this study wanted to know as much as they could about COVID-19, such as the characteristics of the new virus, its mutations and virulence. They made efforts to read and listen to the several sources of information in order to make a cohesive picture about the characteristics of severe acute respiratory syndrome coronavirus 2 (SARS-CoV-2) and the clinical presentation (symptoms) of the infection. Most people wanted to know how the virus was transmitting and spreading, how resistant it was within different materials and how to protect themselves from the virus. Some participants were looking forward to hearing information about the current therapy and vaccination. A few people were not interested in any specific information except when the epidemic would come to an end. The study participants also regularly followed the news about the organization of everyday life during the pandemic.

However, over the course of the epidemic, the interest in all details about the virus began to decrease. Some people got more familiar with COVID-19, while others just felt saturated with information about COVID-19. On the other hand, others were not particularly interested in knowing much about COVID-19 and the pandemic.

“*I was well informed, I wanted to know more about the virus, and I read many articles and I know about it quite well, about its capacity to mutate, as with any virus, but I did not expect that it would take this long because there were other viruses before, and they simply disappeared*.”

(Participant 1)

“*No, it was not my priority at all; I am not an expert in that, so I do not want to deal with it, to be honest*.”

(Participant 10)

#### 3.2.3. Reporting

Within this theme we identified several sub-themes: information access, extent of information, information saturation, lay people in media, inconsistent reporting and the psychological burden of information. Each sub-theme is elaborated.

##### Information Access

All the participants felt that they obtained information about COVID-19 quite easily. In fact, whenever they wanted to get specific pieces of information, they did not have to search for long. Some participants were satisfied with how information about COVID-19 was communicated to the public. They also considered themselves to be adequately informed about the epidemic.

“*I think that our people did it all very well, to be honest. And the very organization of all of those things was at a very high level, given what the others [other countries] were doing*.”

(Participant 9)

“*I think that, for the most part, it was easy to get information, so I think that I am properly informed and well informed*.”

(Participant 4)

##### Extent of Information

Some elderly people in our study felt that the extent of information about COVID-19 was too specific and understandable only to a small group of professionals. For this reason, they considered that the information that was presented in the media exceeded their ability to follow the news in a meaningful manner. They felt that a lack of summarized, clear and straightforward information delivered in a simple and comprehensible way was missing.

“*Look, we are not educated to be able to understand your doctor expressions, so to say*.”

(Participant 9)

“*They talked about it so much, so professionally, that an ordinary person, who does not have a clue about medicine, you know…it’s questionable how well they understood it…I mean, only people who studied that, who had some education related to medicine and health care were able to follow all that*.”

(Participant 11)

The participants tried to filter all the available information to separate what was meaningful to them as well as what they could apply in everyday life. This was especially true for people with little to no understanding of the nature of the virus, who had difficulties following the information. Some participants articulated that they were not interested in knowing additional information a few months into the epidemic, but the information reached them anyway through media because of the abundance of it all around.

“*I just listened to it for my own orientation…because that was what I could grasp…just what was on TV. I did not read anything else, about how it enters the cells…proteins…I was not interested in it…I mean, I should have then to sit down and study it. So I just did not want to study it and get myself all confused*.”

(Participant 8)

##### Information Saturation

With the intense daily influx of information about COVID-19, many participants in our study recalled that they felt overwhelmed with the quantity of it brought to them every day. This was primarily because the information was repetitive and similarly delivered in all media.

“*Listen, it was way too much, because the same thing was always discussed on every television or radio channel*.”

(Participant 8)

“*I was bothered that there was so much talk about it, I mean, wherever you turn to—They talk only about that*.”

(Participant 9)

Moreover, all modes of information delivery were focused on COVID-19, so our participants felt that no other content was considered in the media. Therefore, they felt forced to only think about and discuss issues surrounding COVID-19, as if nothing else was going on besides the epidemic. As a result, many gave up and stopped listening to what was being discussed publicly.

“*I have a remote controller and that is how I solve the problem of too much information or something that I heard before*”

(Participant 3)

“*To be honest, I follow less and less that information. I have reached information overload*.”

(Participant 1)

##### Lay People in Media

Another problem that our participants encountered during the epidemic was the fact that other people, who were not medical experts, were allowed to discuss the epidemic publicly and in the media. Based on the opinion of our participants, this caused a lot of confusion, particularly among people with a lower education level. The fact that people who were not physicians were given space to talk to the masses was deemed inappropriate because it created confusion, doubt, and overall misunderstanding. Overall, the people in our study were bothered when individuals who did not have a medical background were permitted to talk on TV about the virus and the epidemic.

“*…and then [name of the person] was giving us advice. I mean, they do the job that is not theirs to do and it was so disturbing*.”

(Participant 13)

##### Inconsistent Reporting

Many people in this study acknowledged that there were certain discrepancies in expert opinions, particularly about the susceptibility to SARS-CoV-2 and the treatment. The participants acknowledged that the body of knowledge was increasing, albeit at a slow pace, and that recommendations were changing over time. However, their recognition of the COVID-19 epidemic was that there was still a great deal of data that were unclear. The contradictory information and advice were particularly confusing for elderly people, and some of them often did not truly know and understand what was accurate and reliable. A few participants worried about inaccurate or even false information spread by some people (intentionally or unintentionally) mostly on the Internet but also in other media. For this reason, some of them decided to “trust their gut” and use common sense about how to protect themselves from the virus.

“*Well, we always listened to what they were saying on TV. And then came [name of the person]. He began with a different theory…so, we listened to him, but…you had to balance it all. And so, when we started balancing, it means that…it means that you did not listen to anyone, you know…you found your own middle ground…*”

(Participant 8)

“*…And what’s worse—You can find all sorts of information. All sorts of different theories, stories, from the theological ones, you know, ‘God made it’, to those that it was a man-made virus…it has become a hard-core philosophy to me*.”

(Participant 9)

##### Psychological Burden of Information

During the lockdown in Serbia (from March to May 2020) and later on, there were official press conferences from the team that was in charge of prevention and control of the epidemic. The press conferences were held in the early afternoon every working day and occasionally on weekends. This information always included the number of people who were tested, people who were positive, people who were hospitalized and people who were placed on ventilators as well as those who had died in the past day.

Hearing those numbers, especially those who died every day, was particularly distressing for our participants, and this made them feel frustrated because of two reasons: first, they were disturbed by all the numbers they had to listen to, and second, they were frustrated by the number of people who caught the virus, considering them irresponsible for disregarding the advice from medical professionals. Because of all the above-mentioned reasons, the interviewees had troubles thinking of or doing other things that made them feel less stressed. The participants felt that they were bombarded with COVID-19-related information and that they did not have enough mental space to contemplate and process other things in their lives.

“*Too often all of that…every day, every TV station, 50 times this, that…how many people died…it bothered me. Something every day, 50, 320, 33…it was horrific. Whichever channel you turn on they talk about the numbers, so I was switching it off. What do I need those numbers for? You read it in the morning—And that’s it.*”

(Participant 7)

Another issue that was bothersome for our participants was the fact that fatalistic predictions and dramatic announcements related to the forthcoming course of the epidemic were broadcasted on TV. Such pieces of information were especially stressful for elderly people and made them fear and feel tense about the entire situation they were in. As elderly people are generally disciplined in that they follow the official recommendations more strictly than younger population groups, they felt as if their lives were in danger even in their own homes. This information, in fact, made them more anxious.

“*They said that the infection is so severe, that, well, high mortality and so…it, it left, sort of a very unpleasant impression*.”

(Participant 6)

#### 3.2.4. Suggestions for Better Reporting

The participants in this study articulated several ways in which reporting about the epidemic could have been improved. Most of them stated that there should be less information available in the public domain. They also recommended that the information should be delivered at a lower frequency for the lay audience to avoid confusion. Further, it was highlighted that discussion about the epidemic should not be so dramatic, but rather calm, rational and discussed with more tact and that evidence-based and accurate information should be prioritized. The elderly people in this study acknowledged that the key to adequately informing the population was to minimize the errors in reporting so that the flow and direction of the preventative activities was streamlined and less troublesome.

“*They were supposed to be more factual. Because after hearing their reports, I was insane… And I did not know whose opinion to stick to. Still, you always do what you think you should…you listen to them but…again, you want to do what you think…so that it’s easier and you can overcome it, you know*.”

(Participant 8)

“*It should be as convincing and as realistic as it can be, so that there are no errors in any of the statements or something that makes people doubt what they have just heard*.”

(Participant 4)

“*Calmer, tactful, with more scientific details, citing scientific information, research papers, investigations. Absolutely calmer*.”

(Participant 2)

## 4. Discussion

This study intended to bring awareness about information content and delivery related to the COVID-19 epidemic in Serbia. Overall, the elderly people in this study had challenges processing all the pieces of information that reached them and found some of it to be burdensome. While all of them followed the media reports, they were bothered by the extent, repetitiveness, contradictory opinions and unfitting language. The elderly people in this study were also bothered by the fact that people who did not have medical credentials publicly discussed the epidemic. These features made them lose interest in the news. As a result, they made efforts to find their own suitable way to put all the pieces of information together in a picture that was clear and understandable.

The main sources of information for our elderly adults were the TV and the newspapers. Similar results were observed in a qualitative study among middle-aged and elderly African Americans [13] as well as in surveys of elderly Finns [14] and Greeks [15] and the residents of Hong Kong [16] and China [17]. These sources of information can be considered formal because the information delivery is professionally edited and adjusted for a mass audience before it is published. They typically broadcast official information. On the other hand, informal sources of information (various Internet platforms, word of mouth, etc.) may contradict the information published in the formal sources [18] and increase the likelihood of unfavorable outcomes, such as vaccine hesitancy [19,20]. These sources can also be associated with a higher degree of misinformation and lower COVID-19-related knowledge [21,22].

Nevertheless, some participants in this study were hesitant about which sources to trust because they found discrepancies in reporting between different sources, and often the language used to communicate information was inadequate. A study of news content about COVID-19 in Singapore reported that official sources can also deliver erroneous data and contribute to the misinformation of their audience [23]. A particularly burdensome situation was the fact that medical doctors were inconsistent about the news about COVID-19. Because of the rapid influx of new evidence, it may be difficult to keep up with the updates about COVID-19 [24]. On the other hand, it is above all important to avoid discussing unverified information based on assumptions and anecdotes rather than on scientific evidence [24,25]. Critique of the official media by elderly people in Spain due to confusing, deficient and conflicting reporting has been previously published [7]. Furthermore, “a flooding of information” that was also deemed inaccurate was observed in the US [8]. Thus, this phenomenon is not specific to one culture but is likely present worldwide. However, this calls for stricter editing and scrutiny of information delivered from official sources in order to foster public trust [26] and ultimately act for the good of the people.

The quality and quantity of information in media plays a paramount role in how people respond and behave [27,28]. Bearing this in mind, the media should operate in such a way to prevent information overload and fatigue [29] and provide useful and meaningful clues that would have a lasting influence on how elderly people perceive contemporary public health challenges. Thus, communication about the epidemic needs to be coordinated and free from unnecessary and incomprehensible information [30] that distracts the target audience. To help the media improve its information delivery to be both accurate and tempered, a 12-item checklist has been proposed to protect people against infodemics [31]. Such a strategy could be beneficial in efforts to streamline the reporting of potential future epidemics.

### Recommendations

Based on the findings of this study, several recommendations for media reporting during pandemics are in place. First, it is of paramount importance that news outlets adjust the delivery of scientific information so that lay people, with at least a primary education level, are able to understand it. Second, people who do not have relevant credentials should not be granted opportunities to publicly discuss their views because they are not able to adequately tackle the problem. Third, health care systems should enlist authorities to facilitate infodemic control. Fourth, tempered reporting is essential to deliver serious and important pieces of information without generating panic. Conflicting information should be double-checked before broadcasting.

Some limitations of this study should be addressed. The majority of people who participated were highly educated. We did not include people of a lower education level; however, we anticipate that their responses could overlap to a certain extent with the responses observed in this study. Nevertheless, our results cannot be generalized to the entire elderly population of Serbia. We did not test participants’ knowledge on COVID-19, which could have led to a difference in perspectives among the sampled participants. Although the snowball sampling method can introduce a selection bias, it allowed the researchers to openly discuss the research question and record authentic interpretations. Some may argue that the study sample was somewhat small; however, a clear thematic saturation was observed, and many common (sub)themes were acknowledged.

Qualitative studies, such as this one, are necessary to understand new events (such as COVID-19) and nuances in their perceptions, understanding and interpretation. They offer a base on which questionnaires regarding health literacy, knowledge, barriers etc. can be developed and tested on a larger population sample (quantitative studies). For this reason, mixed-method studies (qualitative-quantitative) could be a good methodological tool in the investigation of emergencies in the future.

## 5. Conclusions

In conclusion, the elderly people in Serbia trusted official sources and followed the mainstream media to get information about COVID-19. In this process, they perceived that there were too many pieces of information that were excessively repeated but at the same time conflicting and not coherent enough to match their level of understanding. Confusing messages were facilitated by the presence of people without medical backgrounds in media. These findings should be taken into consideration when delivering health-related information to elderly people.

## Figures and Tables

**Table 1:** Thematic guide for the interviews about processing of COVID-19 information for elderly people.

Topic	Questions
1. Interest and sources of information	(A) Were you interested in knowing everything about the COVID-19 epidemic?
	(B) What information about the COVID-19 pandemic were you most interested in?
	(C) What sources of information did you use to get updated about COVID-19?
	(D) If you stopped following certain sources, why did you stop?
	(E) What sources of information did you believe the most?
2. Difficulties in finding the right information	(A) Was it easy for you to get informed about the COVID-19 epidemic?
	(B) Do you think you were accurately and correctly informed about the epidemic?
	(C) Did you have any difficulties in grasping all the information that was out there?
3. Current feelings and behaviors	(A) From this standpoint, do you think that information delivery should have been different?
	(B) In case of a new epidemic, what would you consider to be optimal reporting?

## Data Availability

Full transcripts are available on a reasonable request to the corresponding author.
